# Mild Traumatic Brain Injury and Criminal Charges and Convictions in Mid and Late Adolescence

**DOI:** 10.1001/jamapediatrics.2024.3452

**Published:** 2024-09-30

**Authors:** Ea Hoppe Blaabæk, Daniel Juhász Vigild, Felix Elwert, Peter Fallesen, Lars H. Andersen

**Affiliations:** 1 ROCKWOOL Foundation, Copenhagen, Denmark; 2 Department of Sociology, University of Copenhagen, Copenhagen, Denmark; 3 Copenhagen Center for Social Data Science, University of Copenhagen, Copenhagen, Denmark; 4 Department of Sociology, University of Wisconsin–Madison; 5 Department of Biostatistics and Medical Informatics, School of Medicine and Public Health, University of Wisconsin–Madison; 6 Department of Population Health Sciences, School of Medicine and Public Health, University of Wisconsin–Madison; 7 Swedish Institute for Social Research, Stockholm University, Stockholm, Sweden

## Abstract

**Importance:**

Childhood exposure to mild traumatic brain injury (mTBI) is common. Individuals with a childhood history of mTBI experience more frequent criminal justice involvement in mid to late adolescence and adulthood. No study had been conducted to examine whether the link is causal or spurious.

**Objective:**

To determine whether mTBI in childhood causes criminal justice involvement in mid to late adolescence.

**Design, Setting, and Participants:**

This cohort study used population-based data for all children born between 1995 and 2000 in Denmark, with data linked to emergency department (ED) visits and hospitalizations before age 10 years and all criminal charges and convictions from ages 15 to 20 years. The exposure group contained all individuals diagnosed with mTBI before age 10 years without other intracranial or extracranial injuries; the comparison group was individuals not diagnosed with mTBI or intracranial or extracranial injuries. Sibling and twin fixed-effects models were used to evaluate the association after controlling for family-level confounding. Data were analyzed from May 2021 to July 2024.

**Exposures:**

Mild TBI before age 10 years without other intracranial or extracranial injuries before or at the time of diagnosis.

**Main Outcomes and Measures:**

Associations between mTBI before age 10 years and criminal charges and convictions from ages 15 to 20 for the entire study population and separately by sex at birth, controlling for additional covariates.

**Results:**

The final analytic sample consisted of 343 027 individuals, 13 514 in the exposure group and 329 513 in the comparison group. Of the total sample, 166 455 (49%) were female and 176 572 were male (51%). A total of 326 191 participants (95%) had at least 1 parent with Danish citizenship, and 79 386 mothers (23%) held a college degree. There was a positive association between mTBI and criminal charges (odds ratio [OR], 1.26; 95% CI, 1.19-1.34) and convictions (OR, 1.24; 95% CI, 1.16-1.33). When controlling for family-level confounding, the associations became statistically insignificant and, in most models, greatly reduced. Results were robust across multiple model specifications.

**Conclusions and Relevance:**

This study found that although mTBI in childhood was predictive of adolescent criminal justice involvement, there was no evidence that mTBI caused criminal charges or convictions.

## Key Points

**Question:**

Does mild traumatic brain injury (mTBI) before age 10 years cause criminal charges and convictions in mid to late adolescence (ages 15-20 years)?

**Findings:**

This cohort study found a strong positive association between mTBI in childhood and criminal charges and convictions in adolescence among 343 027 individuals in Denmark. However, the associations nearly disappeared and became statistically insignificant when accounting for family-level confounding factors.

**Meaning:**

There is no evidence that childhood mTBI causes criminal charges or convictions in mid to late adolescence.

## Teaser

This cohort study examines population data for Denmark to determine any associations between childhood mild traumatic brain injury (occurring before age 10 years) and criminal charges and convictions at ages 15 to 20 years.

## Introduction

Concussions and mild traumatic brain injuries (mTBIs) are among the most common childhood injuries. An estimated 4% of children experience an mTBI before age 10 years,^1^ of which 25% experience persistent postconcussion symptoms.^2^ The positive association between general head injuries and antisocial behavior is well established.^3,4,5,6,7,8,9^ Specifically, mTBI is associated with increased behavioral problems, mental health disorders, and aggression in children.^10,11,12,13^ Young people with a history of mTBI are greatly overrepresented among those with a history of criminal justice involvement. Estimates of ever having experienced an mTBI among incarcerated and criminally active youth range between 30% and 70%.^14,15,16^ Because of its strong positive associations with poor mental health and antisocial behavior, mTBI has been hypothesized to cause criminal offending.^3,7,13,17^

However, it remains unclear whether the positive association between mTBI and criminal offending is causal or spurious due to confounding by other, possibly unobserved, background characteristics. On one hand, supporting a causal interpretation, mTBI is known to disrupt axonal connectivity in the brain,^18,19^ thereby diminishing attentional control and inhibitory functions^20,21^ and increasing the risk of impulsive aggression, poor decision making, and ultimately criminal behavior.^4^ On the other hand, supporting a spurious association, some children grow up in disadvantaged home environments and neighborhoods, which may predispose them to mTBI exposure and criminal behavior.^22,23,24^

We study the association between mTBI exposure and criminal charges and convictions in Denmark, where mTBIs account for more than 80% of all incidences of head trauma^25^ at a rate of 457 per 100 000 people each year.^26^ We analyzed longitudinal population-level data on all individuals born between 1995 and 2000 in Denmark linked to their individual hospitalization and criminal justice histories. To avoid reverse causality, we focus on mTBI exposure occurring before age 10 years and criminal justice involvement when aged 15 to 20 years. To address confounding in the association between mTBI and criminal justice involvement, we present sibling and twin comparisons (fixed-effect models), which control for observed and unobserved shared risk factors for mTBI and criminal justice involvement at the family level.

## Methods

### Ethics and Preregistration

We followed a detailed preanalysis plan, preregistered at OSF Registries.^27^ Because we analyzed pseudo-anonymized Danish registers^28^ in compliance with the EU General Data Protection Regulation, no internal review board approval or participant consent was required for this study under the Law of Statistics Denmark.^29^ We followed Strengthening the Reporting of Observational Studies in Epidemiology (STROBE) reporting guideline.

### Data

**Figure 1.**
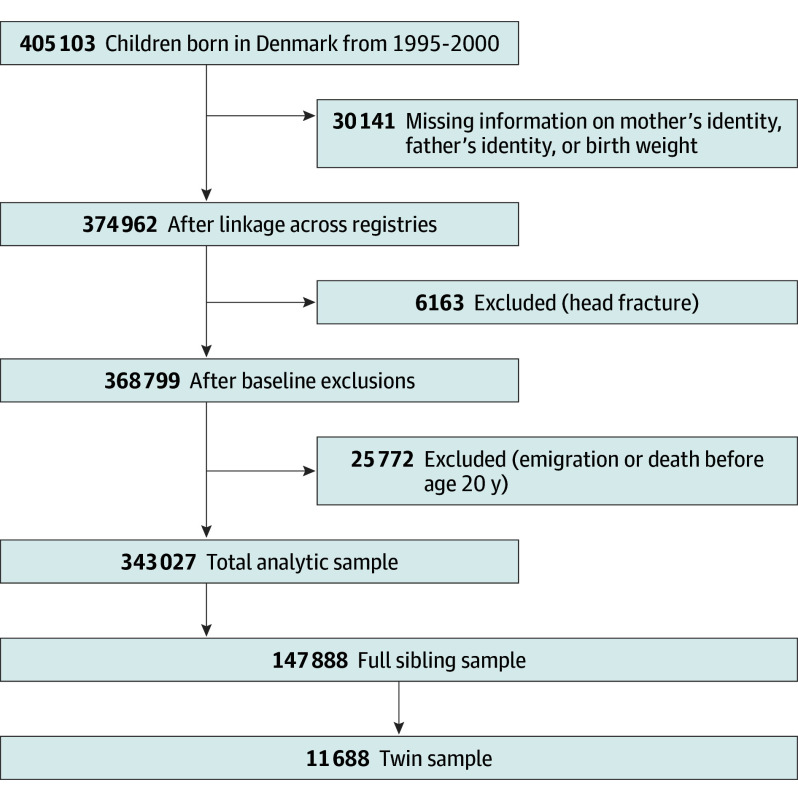
Sample Construction Flow diagram for sample construction.

We compiled our analytic data from administrative population registers in Denmark that were linked using unique personal identifiers (Figure 1). Sample constructions starts from all individuals born in Denmark between 1995 and 2000 (N = 405 103).^30^ We excluded individuals without father ID, mother ID, or birth weight and individuals who died or emigrated before their 20th birthday.^30^ We also excluded individuals who had a head, neck, or brain injury (ascertained from the Danish National Patient Register^31,32^ and *International Statistical Classification of Diseases and Related Health Problems, Tenth Revision *[*ICD-10*] codes S02, S04, S06, S07, and S12) in or before the year of their first mTBI if they had an mTBI or ever if they did not have an mTBI. We did this to isolate the association between mTBI and criminal charges and convictions from the consequences of more severe TBIs or other head injuries that often co-occur with mTBIs. Twins were defined as 2 full siblings born within 2 consecutive days. Data were analyzed from May 2021 to July 2024.

### Variables

#### Exposure Variable

We coded a binary indicator variable for ever being diagnosed with an mTBI in an emergency department (ED) or hospital between birth and age 10 years (1 = yes, 0 = no) from the Danish National Patient Register^31,32^ We defined mTBI as *ICD-10* code S06.0 (intracranial injuries without open intracranial wounds).^31^ We focused on mTBIs in childhood to avoid reverse causality (mTBI arising from criminal behavior) and because head injuries before the internalization of social and moral behavior are more likely to result in antisocial and aggressive behavior.^3^

#### Outcome Variables

We coded 2 binary indicator variables (1 = yes, 0 = no) for whether a sample member was ever charged with a crime (by a public prosecutor) or ever convicted (in a precourt settlement or an in-court ruling) between ages 15 and 20 years. We obtained this information from Danish Criminal Justice Registers that contain the universe of formal charges and convictions.^33,34^ Age 15 years is the age of criminal liability, and age 20 years is the peak risk of criminal conviction in Denmark.^35^ Criminal charges and convictions comprise violations of the Danish Penal Code, which excludes minor offenses, such as traffic violations and minor drug possession. We also analyzed criminal charges and convictions by crime type (all destructive crimes [eg, vandalism, violence] and violent crimes against people only [eg, assault]). In supplementary analyses, we reanalyzed all models using the number of charges or convictions.

#### Control Variables

To address observed confounding, we controlled for binary sex ascribed at birth, year of birth (categorical: yearly increments), birth weight (categorical: above/below 2500 g), birth order in the full sibship (categorical: 1-5), mother’s and father’s educational attainment (categorical: less than high school, high school/vocational training, college), and parents’ citizenship status at child’s birth (categorical: at least 1 parent had Danish citizenship, neither parents were Danish citizens and at least 1 parent was born in or was a citizen of a western country, neither parent was a Danish citizen and both parents were born in or were citizens of nonwestern countries). Coding of the citizenship variable follows the conventions of Statistics Denmark.^36^ The supplement shows additional preregistered exploratory model specifications, which show that results are robust to (1) entering birth weight as linear, linear and quadratic, or log-transformed terms (eTables 1-3 in Supplement 1 ) and (2) controlling only for sex at birth and year of birth (eTable 4 in Supplement 1).

### Statistical Models

We analyzed 3 samples in the targeted birth cohorts: all individuals, all full siblings (sharing the same biological father and mother), and all twins. Our analysis proceeded in 6 steps. First, we presented descriptive statistics. Second, we compared the risk of criminal charges and convictions by mTBI status separately by crime type (all crimes, destructive crimes, and violent crimes) and by sex at birth. Third, we adjusted for observed confounding between mTBI and criminal charges and convictions by controlling for observed covariates in logistic regression models.

Fourth, we controlled for unobserved confounding by estimating sibling and twin fixed-effect models using conditional logistic regression.^37^ Sibling fixed-effects models compared individuals who were diagnosed with an mTBI before age 10 years to a full sibling without an mTBI diagnosis and thus control for siblings’ shared characteristics, such as the shared home environment, neighborhood, and parental characteristics, as well as, on average, 50% of their genetic makeup. Comparing twins allows for an even more closely matched comparison because twins typically share their entire childhood environment and 50% to 100% of their genetic makeup. Therefore, whereas the analysis of the entire sample is likely confounded by unobserved risk factors, confounding bias is better controlled in the sibling analysis and best controlled in the twin analysis. (Per the data use agreement with Statistics Denmark, we do not report results from twin fixed-effect models for females because of small cell sizes.^38^)

We assessed the credibility of our sibling and twin fixed-effect models by implementing a falsification test. The key assumption of our sibling and twin fixed-effects models is that mTBI is as-if randomly assigned within sibships net of observed sibling-varying covariates.^37,39^ This assumption would be challenged if the sibling experiencing the mTBI also experienced a systematically higher risk of sustaining other childhood injuries. Therefore, we tested the association between mTBI and other injuries within sibships and would interpret a statistically significant association as a challenge to the fixed-effects assumption.

Fifth, to test our results’ sensitivity to outcome definitions, we estimated separate models for crime categories (destructive and violent crimes) and by sex at birth. Sixth, to assess robustness to model choice, we reran all models using ordinary least squares (OLS) regression for binary outcomes and Poisson regression for the number of criminal charges or convictions.

We present point estimates (odds ratios [ORs] for logistic regression, risk ratios for Poisson regression, and linear coefficients for OLS) and 95% CIs. All tests are 2-tailed. Standard errors are clustered at the family level. All statistical analyses were conducted in R version 4.3.1 (R Foundation).^40,41,42^

## Results

The final analytic sample consisted of 343 027 individuals, of whom 147 888 (43.1%) were full siblings and 11 688 (3.4%) were twins. Across samples, 4% were diagnosed with an mTBI before age 10 years, 5% to 6% were charged with a crime, and 4% to 5% were convicted of any crime prior to age 20 years (eTable 5 in Supplement 1). The risk of being diagnosed with an mTBI was highest between ages 0 and 4 years for males and females and declined gradually thereafter (eFigure 1 in Supplement 1). Table 1 presents descriptive demographic statistics. Of the total sample, 166 455 (49%) were female and 176 572 were male (51%). A total of 326 191 participants (95%) had at least 1 parent with Danish citizenship, and 79 386 mothers (23%) held a college degree. Descriptive statistics are comparable across samples, except that twins were more likely to have low birth weight (<2500 g; 5% for all vs 40% for twins). (eTable 5 in Supplement 1 additionally shows descriptive statistics for outcome variables.)

**Table 1.** Demographic Indicators Across Total, Sibling, and Twin Samples

Characteristic	No. (%)		
	Total sample (n = 343 027)	Sibling sample (n = 147 888)	Twin sample (n = 11 688)
Sex at birth			
Female	166 455 (49)	71 854 (49)	5706 (49)
Male	176 572 (51)	76 034 (51)	5982 (51)
Parent citizenship^a^			
Danish	326 191 (95)	139 501 (94)	11 281 (97)
Western country	1109 (0.3)	419 (0.3)	30 (0.3)
Nonwestern country	15 727 (5)	7968 (5)	377 (3)
Mother’s education			
Less than high school	87 705 (26)	33 645 (23)	2598 (22)
High school and vocational	175 936 (51)	76 820 (52)	6103 (52)
College	79 386 (23)	37 423 (25)	2987 (26)
Father’s education			
Less than high school	88 044 (26)	34 335 (23)	2700 (23)
High school and vocational	192 774 (56)	84 461 (57)	6710 (57)
College	62 209 (18)	29 092 (20)	2278 (19)
Birth order			
1	166 664 (49)	56 932 (38)	3330 (28)
2	125 242 (37)	67 682 (46)	5204 (45)
3	40 096 (12)	17 569 (12)	2376 (20)
4	8160 (2)	4129 (3)	599 (5)
5	2865 (1)	1576 (1)	179 (2)
Birth year			
1995	59 443 (17)	24 989 (17)	1838 (16)
1996	57 325 (17)	23 715 (16)	1996 (17)
1997	56 791 (17)	24 904 (17)	1943 (17)
1998	56 139 (16)	25 196 (17)	1951 (17)
1999	56 214 (16)	23 996 (16)	1922 (16)
2000	57 115 (17)	25 088 (17)	2038 (17)
Birth weight >2500 g	326 402 (95)	139 150 (94)	7035 (60)

^a^ Citizenship was a categorical variable: at least 1 parent had Danish citizenship, neither parents were Danish citizens and at least 1 parent was born in or was a citizen of a western country, or neither parent was a Danish citizen and both parents were born in or were citizens of nonwestern countries.

Table 2 shows unadjusted descriptive rates of criminal charges and convictions (by mTBI, crime type, and sex). Individuals diagnosed with an mTBI before age 10 years were more likely to have been charged with or convicted of a crime before age 20 years than those who were not diagnosed with mTBI (8.4% vs 6.3% for charges; 6.6% vs 5.0% for convictions). Therefore, the excess risk of criminal charges is 33% in the mTBI group, and the excess risk of criminal conviction is 32% in the mTBI group. Males are more likely to be charged with and convicted of crimes than females in general and across crime types.

**Table 2.** Rate of Criminal Charges and Convictions by mTBI Status, Crime Type, and Sex^a^

Crime type	Sample	Rate (95% CI), %	
		No mTBI	mTBI
**Charged**			
All crimes	Males and females	6.3 (6.2-6.4)	8.4 (7.9-8.9)
	Males	9.0 (8.9-9.2)	11.6 (10.9-12.3)
	Females	3.4 (3.3-3.5)	4.2 (3.7-4.7)
Destructive	Males and females	3.2 (3.2-3.3)	4.6 (4.2-4.9)
	Males	5.4 (5.3-5.5)	7.1 (6.5-7.6)
	Females	0.9 (0.9-1.0)	1.4 (1.1-1.7)
Violent	Males and females	1.8 (1.8-1.9)	2.8 (2.5-3.0)
	Males	3.0 (2.9-3.1)	4.2 (3.7-4.6)
	Females	0.6 (0.6-0.7)	0.9 (0.7-1.2)
**Convicted**			
All crimes	Males and females	5.0 (4.9-5.1)	6.6 (6.2-7.0)
	Males	7.1 (6.9-7.2)	9.0 (8.3-9.6)
	Females	2.8 (2.7-2.9)	3.5 (3.1-4.0)
Destructive	Males and females	2.2 (2.1-2.2)	3.0 (2.7-3.3)
	Males	3.6 (3.5-3.7)	4.7 (4.2-5.1)
	Females	0.6 (0.6-0.7)	0.9 (0.6-1.1)
Violent	Males and females	1.2 (1.2-1.3)	1.8 (1.6-2.0)
	Males	2.0 (1.9-2.1)	2.8 (2.4-3.1)
	Females	0.4 (0.4-0.5)	0.6 (0.4-0.8)

Abbreviation: mTBI, mild traumatic brain injury.

^a^ Unadjusted share (and 95% CI) of participants who have ever been charged and/or convicted of a crime when 15 to 20 years old across mTBI status (before age 10 years), crime type, and sex.

Table 3 shows estimates for the association between mTBI before age 10 years and being charged with or convicted of a crime between age 15 and 20 years net of observed covariates with and without sibling and twin fixed effects. When we controlled only for observed covariates, the odds ratios for the associations between mTBI and being charged with a crime (OR, 1.26; 95% CI, 1.19-1.34 [model 1]) and between mTBI and being convicted of a crime (OR, 1.24; 95% CI, 1.16-1.33 [model 4]) were positive and statistically significant.

**Table 3.** Associations Between mTBI and Being Criminally Charged and Convicted for All Participants, Siblings, and Twins^a^

Characteristic	Charged			Convicted		
	Model 1	Model 2	Model 3	Model 4	Model 5	Model 6
mTBI risk, OR (95% CI)^b^	1.26 (1.19-1.34)	0.97 (0.85-1.10)	0.94 (0.58-1.53)	1.24 (1.16-1.33)	0.95 (0.82-1.09)	0.84 (0.51-1.41)
Sample type	Total	Siblings	Twins	Total	Siblings	Twins
Family fixed effects	No	Yes	Yes	No	Yes	Yes
No. of participants	343 027	147 888	11 688	343 027	147 888	11 688

Abbreviations: mTBI, mild traumatic brain injury; OR, odds ratio.

^a^ Estimates for the association between mTBI before age 10 years and ever being charged with or convicted of a crime when 15 to 20 years old.

^b^ ORs and 95% CIs for the association between mTBI and criminal charges and convictions from logistic regression models (without fixed effects) and conditional logistic regression models (with fixed effects). Standard errors were clustered at the family level. Control variables included sex at birth, year of birth, birth weight, birth order, parents’ educational levels, and parents’ citizenship.

Adding sibling fixed effects to control for unobserved confounding substantially reduced the magnitude of the associations (OR, 0.97; 95% CI, 0.85-1.10, for charges [model 2]; OR, 0.95; 95% CI, 0.82-1.09, for convictions [model 5]), which were no longer statistically significant. Twin fixed-effects models yielded point estimates of similar size but with much larger standard errors due to the smaller sample size (OR, 0.94; 95% CI, 0.58-1.53, for charges [model 3]; OR, 0.84; 95% CI, 0.51-1.41, for convictions [model 6]).

**Figure 2.**
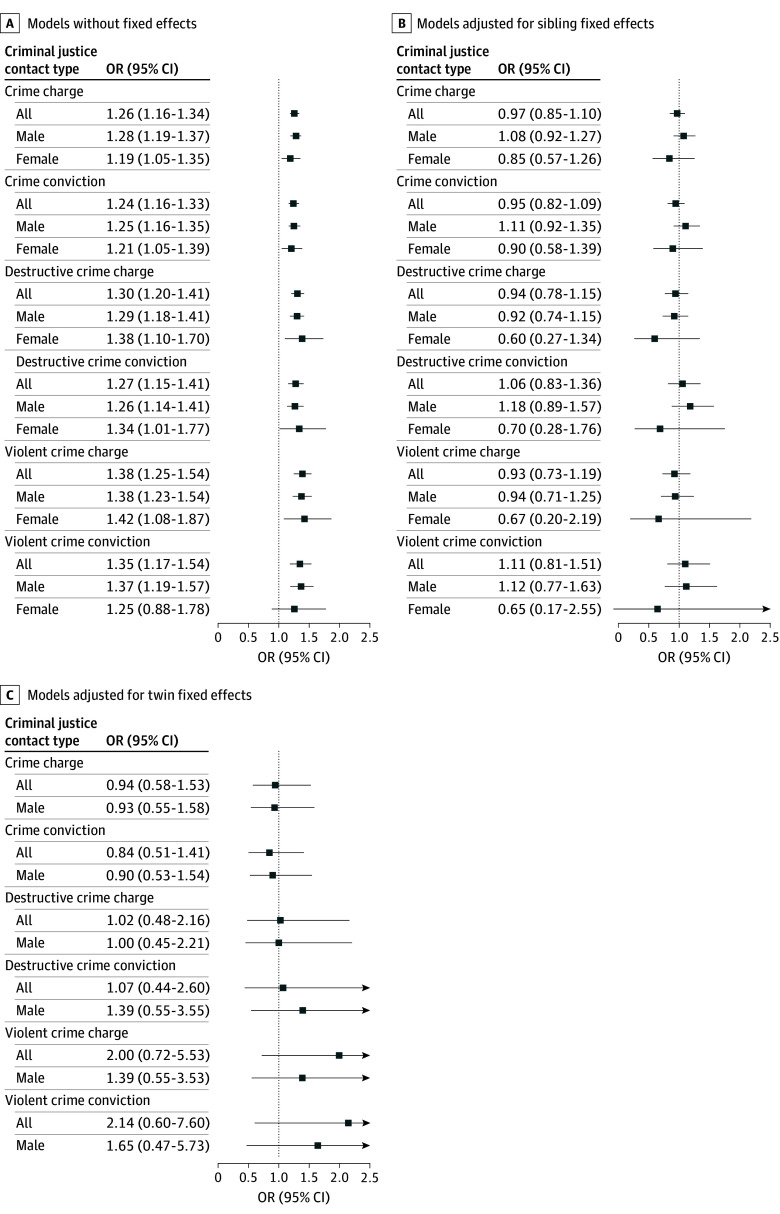
Association Between Mild Traumatic Brain Injury and Crime Across Crime Type and Sex at Birth The figure shows odds ratios for the association between mild traumatic brain injury before age 10 years and ever being charged with or convicted of a crime when aged 15 to 20 years across 3 model specifications and nested samples. Results for females in twin fixed-effects models are masked because of insufficient cell size (a confidentiality constraint imposed by Statistics Denmark).

Figure 2 illustrates the robustness of our estimates across model specifications. Specifically, the positive, substantively large, and statistically significant associations between mTBI and criminal charges and convictions found in the samples of all individuals, males, and females were reduced in magnitude (in almost all models) and became statistically insignificant (in all models) when we controlled for unobserved confounding using sibling or twin fixed effects.

We assessed the credibility of the fixed-effect assumption by estimating sibling and twin fixed-effect models for the within-sibling/-twin correlations between mTBI and other injuries (excluding injuries co-occurring with the mTBI). eTable 6 in Supplement 1 shows little evidence of statistically significant associations. The only exception is that siblings who experienced an mTBI had 0.04 more ED visits with minor contusions (eg, cuts, bruises) before age 10 years, which we judged substantively unimportant. Therefore, we found no evidence of substantial violations of the fixed-effect assumption.

We found similar results using OLS and Poisson models (eFigures 2-5 in Supplement 1) for binary and count outcomes. eTables 7-15 in Supplement 1 report detailed estimates for all specifications illustrated in Figure 2 and eFigures 2-5 in Supplement 1.

## Discussion

Research has shown that mTBI and TBI are positively associated with greater criminal and risk-seeking behavior,^4,5,6,7,8,9^ and individuals ever diagnosed with mTBI and TBI are overrepresented in populations of offenders.^14,15,16^ These associations implicate mTBI in criminogenesis, suggesting that mTBI may be causally responsible for crime.^3,7,13,17^ We conducted the largest study of mTBI, criminal charges, and criminal convictions to date, analyzing longitudinal data on nearly 350 000 Danish individuals linked to complete diagnostic records from ED and hospital visits during childhood and complete criminal justice records from ages 15 to 20 years. Our analysis confirmed that experiencing mTBI during childhood is positively associated with criminal charges and convictions in mid to late adolescence. However, we also found that the positive associations between mTBI and criminal charges and convictions strongly decreased and were no longer statistically significant when we controlled for unobserved social, familial, and genetic factors shared between biological siblings and twins. These results suggest that mTBI in individuals younger than 10 years does not cause criminal behavior in mid to late adolescence and that interventions designed to reduce mTBI in individuals are unlikely to directly reduce criminal offending later on.

### Limitations

We discuss 4 potential limitations. First, sibling and twin fixed-effect designs do not control for unobserved differences between siblings (eg, in personality traits, mental well-being, lifestyle, and peer groups) that may cause mTBI and criminal offending. Such sibling-varying unobserved confounding (eg, due to differential risk seeking) would plausibly induce a positive association between mTBI and criminal offending, such that accounting for it would further reduce our estimates. Therefore, this potential bias, if present, would further support our conclusion of no evidence of a causal link between mTBI and criminal justice involvement. In eTable 6 in Supplement 1, we additionally show that siblings diagnosed with an mTBI before age 10 years have not visited an ED or hospital with fractures or sprains more often than nondiagnosed individuals (and only have slightly more ED visits related to contusions/minor injuries). This supplementary finding also indicates that individuals diagnosed with an mTBI do not exhibit greater risk taking once family-level confounding is controlled.

Second, sibling and twin-fixed effect estimation assumes the absence of between-sibling spillovers; that is, that the exposure and/or outcome of one sibling does not affect the exposure and/or outcome of the other.^43^ However, the presence of most spillover scenarios does not impinge on our main conclusion. First, if one sibling’s mTBI affects the chance of an mTBI in the other (so-called exposure-to-exposure spillover [perhaps because mTBI in the first sibling increases vigilance and reduces risk taking in the other]), our methods would not be biased.^43^ Second, outcome-to-exposure spillover, whereby the criminal justice involvement of one sibling causes the other to suffer an increased or decreased risk of mTBI, would bias our results in an indeterminate direction.^43^ This possibility, however, is ruled out by design in our twin fixed-effect models because outcomes are ascertained 5 or more years after treatments, so one twin’s outcome cannot affect the other’s exposure. Similarly, outcome-to-exposure spillover between siblings is essentially ruled out in sibling fixed-effects models because siblings in the 1995-2000 cohorts can be at most 6 years apart in age and only 3% of siblings differ by 5 years or more years in age (mean birth spacing is 2.6 years). Third, although exposure-to-outcome spillover would bias our estimates toward zero^43^ and outcome-to-exposure spillover would bias our point estimates in an a priori indeterminate direction,^43^ neither scenario would impinge on the ability of our sibling or twin fixed-effects models to test the null hypothesis of no association,^43^ and therefore does not cast doubt on our conclusion of no evidence of a robust association of mTBI and criminal justice involvement.

Third, we excluded more severe forms of TBI from our exposure variable to isolate the unique effect of milder forms of TBIs. This exclusion allowed us to focus on mTBIs, which are more common in the population than TBIs, and hence may have larger cumulative consequences if proven criminogenic. Our results do fail to confirm the existence of such criminogenic effects of mTBIs once family factors are controlled, but more severe forms of TBIs could still have criminogenic effects.^9,44^

Last, the administrative registers did not contain information about undiagnosed mTBIs or mTBIs diagnosed by general practitioners outside of EDs or hospitals. If individuals who are more likely to engage in criminal activity are less likely to go to the ED or hospital for minor injuries or accidents, this would underestimate the association between mTBI and criminal charges and convictions in models that do not control for family-level fixed effects. However, under the assumption that the propensity to go to the ED/hospital is constant across siblings within families, differential mTBI and crime risks across families would not lead to bias in our sibling and fixed-effect models.

## Conclusions

This study documents that experiencing an mTBI in childhood is positively associated with criminal justice involvement in mid to late adolescence in the general population and that this association greatly decreases and becomes statistically insignificant once family-level confounding is controlled. Therefore, previously reported positive associations that do not control for family-level confounding likely overstate the causal effect of mTBI on crime.^7,13,14,15,16^ Interventions to reduce mTBI rates in children are unlikely to reduce crime rates, although they may have other health and social benefits, and more severe forms of TBIs could still have criminogenic effects.^9,44^ Policymakers aiming to decrease crime should view childhood mTBI histories as predictive of rather than causally contributing to adolescent criminal behavior.

## Notes

Supplement 1. **eTable 1.** Robustness to Birthweight Specifications OLS. Outcome: Ever Convicted of a Crime. Models without Fixed Effects **eTable 2.** Robustness to Birthweight Specifications OLS. Outcome: Ever Convicted of a Crime. Models With Sibling Fixed Effects **eTable 3.** Robustness to Birthweight Specifications OLS. Outcome: Ever Convicted of a Crime. Models With Twin Fixed Effects **eTable 4.** Odds Ratios From logistic Regression Models for the Association between Minor Traumatic Brain Injury (mTBI; before age 10) and Ever Being Convicted of a Crime Before Age 20 **eTable 5.** Descriptive Statistics **eTable 6.** Association Between mTBI and the Number of ER Fracture/Sprain/Contusion Events **eTable 7.** OR Estimates From All logit Models on Ever Being Charged or Convicted of a Crime **eTable 8.** IRR Estimates from Poisson Models on Crime Charges and Convictions **eTable 9.** Estimates From OLS Models on Crime Charges and Convictions **eTable 10.** OR Estimates From logit Models on Ever Being Charged or Convicted of a Destructive Crime **eTable 11.** IRR Estimates From Poisson Models on Destructive Crime Charges and Convictions **eTable 12.** Estimates From OLS Models on Destructive Crime Charges and Convictions **eTable 13.** OR Estimates From logit Models on Ever Being Charged or Convicted of a Violent Crime **eTable 14.** IRR Estimates from Poisson Models on Violent Crime Charges or Convictions **eTable 15.** Estimates From OLS Models on Violent Crime Convictions **eFigure 1.** mTBI Rates by Age and Sex **eFigure 2.** Estimated Associations (OLS Models on Binary Outcome) between mTBI (Before Age 10) and Ever Being Charged or Convicted of Criminal Offenses (Age 15 to 20) by Crime Type and Sex Across Preregistered Models **eFigure 3.** Estimated Associations (OLS Models) between mTBI (Before Age 10) and Number of Criminal Charges and Convictions (Age 15 to 20) by Crime Type and Sex Across Preregistered Models **eFigure 4.** Estimated Associations (Incidence Rate Ratios From Poisson Models) between mTBI (Before Age 10) and Ever Being Charged or Convicted of Criminal Offenses (Age 15 to 20) by Crime Type and Sex Across Preregistered Models **eFigure 5.** Estimated Associations (Incidence Rate Ratios From Poisson Models) between mTBI (Before Age 10) and Number of Criminal Charges and Convictions (Age 15 to 20) by Crime Type and Sex Across Preregistered Models

Supplement 2. Data sharing statement
