# Supplementary material for: Mild Traumatic Brain Injury and Criminal Charges and Convictions in Mid and Late Adolescence
Source: JAMA Pediatr. 2024 Sep 30;178(11):1164–71. doi: 10.1001/jamapediatrics.2024.3452 (PMC11443431; doi:10.1001/jamapediatrics.2024.3452)
Supplement: Supplement 1. — eTable 1. Robustness to Birthweight Specifications OLS. Outcome: Ever Convicted of a Crime. Models without Fixed Effects eTable 2. Robustness to Birthweight Specifications OLS. Outcome: Ever Convicted of a Crime. Models With Sibling Fixed Effects eTable 3. Robustness to Birthweight Specifications OLS. Outcome: Ever Convicted of a Crime. Models With Twin Fixed Effects eTable 4. Odds Ratios From logistic Regression Models for the Association between Minor Traumatic Brain Injury (mTBI; before age 10) and Ever Being Convicted of a Crime Before Age 20 eTable 5. Descriptive Statistics eTable 6. Association Between mTBI and the Number of ER Fracture/Sprain/Contusion Events eTable 7. OR Estimates From All logit Models on Ever Being Charged or Convicted of a Crime eTable 8. IRR Estimates from Poisson Models on Crime Charges and Convictions eTable 9. Estimates From OLS Models on Crime Charges and Convictions eTable 10. OR Estimates From logit Models on Ever Being Charged or Convicted of a Destructive Crime eTable 11. IRR Estimates From Poisson Models on Destructive Crime Charges and Convictions eTable 12. Estimates From OLS Models on Destructive Crime Charges and Convictions eTable 13. OR Estimates From logit Models on Ever Being Charged or Convicted of a Violent Crime eTable 14. IRR Estimates from Poisson Models on Violent Crime Charges or Convictions eTable 15. Estimates From OLS Models on Violent Crime Convictions eFigure 1. mTBI Rates by Age and Sex eFigure 2. Estimated Associations (OLS Models on Binary Outcome) between mTBI (Before Age 10) and Ever Being Charged or Convicted of Criminal Offenses (Age 15 to 20) by Crime Type and Sex Across Preregistered Models eFigure 3. Estimated Associations (OLS Models) between mTBI (Before Age 10) and Number of Criminal Charges and Convictions (Age 15 to 20) by Crime Type and Sex Across Preregistered Models eFigure 4. Estimated Associations (Incidence Rate Ratios From Poisson Models) between mTBI (Before Age 10) and Ever Being Charge [file jamapediatr-e243452-s001.pdf]

Supplemental Online Content

Blaabæk EH, Vigild DJ, Elwert F, Fallesen P, Andersen LH. Mild traumatic brain injury and criminal charges and convictions in mid and late adolescence. *JAMA Pediatr*. Published online September 30, 2024. doi:10.1001/jamapediatrics.2024.3452

eTable 1. Robustness to Birthweight Specifications OLS. Outcome: Ever Convicted of a Crime. Models without Fixed Effects
eTable 2. Robustness to Birthweight Specifications OLS. Outcome: Ever Convicted of a Crime. Models With Sibling Fixed Effects
eTable 3. Robustness to Birthweight Specifications OLS. Outcome: Ever Convicted of a Crime. Models With Twin Fixed Effects
eTable 4. Odds Ratios From logistic Regression Models for the Association between Minor Traumatic Brain Injury (mTBI; before age 10) and Ever Being Convicted of a Crime Before Age 20
eTable 5. Descriptive Statistics
eTable 6. Association Between mTBI and the Number of ER Fracture/Sprain/Contusion Events
eTable 7. OR Estimates From All logit Models on Ever Being Charged or Convicted of a Crime
eTable 8. IRR Estimates from Poisson Models on Crime Charges and Convictions
eTable 9. Estimates From OLS Models on Crime Charges and Convictions
eTable 10. OR Estimates From logit Models on Ever Being Charged or Convicted of a Destructive Crime
eTable 11. IRR Estimates From Poisson Models on Destructive Crime Charges and Convictions
eTable 12. Estimates From OLS Models on Destructive Crime Charges and Convictions
eTable 13. OR Estimates From logit Models on Ever Being Charged or Convicted of a Violent Crime
eTable 14. IRR Estimates from Poisson Models on Violent Crime Charges or Convictions
eTable 15. Estimates From OLS Models on Violent Crime Convictions
eFigure 1. mTBI Rates by Age and Sex
eFigure 2. Estimated Associations (OLS Models on Binary Outcome) between mTBI (Before Age 10) and Ever Being Charged or Convicted of Criminal Offenses (Age 15 to 20) by Crime Type and Sex Across Preregistered Models
eFigure 3. Estimated Associations (OLS Models) between mTBI (Before Age 10) and Number of Criminal Charges and Convictions (Age 15 to 20) by Crime Type and Sex Across Preregistered Models
eFigure 4. Estimated Associations (Incidence Rate Ratios From Poisson Models) between mTBI (Before Age 10) and Ever Being Charged or Convicted of Criminal Offenses (Age 15 to 20) by Crime Type and Sex Across Preregistered Models
eFigure 5. Estimated Associations (Incidence Rate Ratios From Poisson Models) between mTBI (Before Age 10) and Number of Criminal Charges and Convictions (Age 15 to 20) by Crime Type and Sex Across Preregistered Models

This supplemental material has been provided by the authors to give readers additional information about their work.

© 2024 Blaabæk EH et al. *JAMA Pediatrics.*

eTable 1. Robustness to Birthweight Specifications OLS. Outcome: Ever Convicted of a Crime. Models without Fixed Effects

	M1	M2	M3	M4
mTBI	0.014***<br>(0.002)	0.014***<br>(0.002)	0.013***<br>(0.002)	0.014***<br>(0.002)
Birth weight kg linear	-0.007***<br>(0.001)	0.020***<br>(0.003)		
Birth weight kg squared		-0.004***<br>(0.000)		
log(birth weight)			-0.020***<br>(0.002)	
Birth weight above/below 2500 g.				0.000<br>(0.002)
FE	None	None	None	None
Observations	343 027	343 027	343 027	343 027

Note: (Standard errors). Estimates and standard errors from OLS models for the association between minor traumatic brain injuries (mTBIs) and ever being convicted of a crime before age 20. Standard errors are clustered at the family level in all models. Control variables: sex and year of birth. Two-sided hypothesis tests, *** P < .001, **P < .01, *P < .05

eTable 2. Robustness to Birthweight Specifications OLS. Outcome: Ever Convicted of a Crime. Models With Sibling Fixed Effects

	M1	M2	M3	M4
mTBI	-0.002<br>(0.004)	-0.002<br>(0.004)	-0.002<br>(0.004)	-0.002<br>(0.004)
Birth weight kg linear	0.003*<br>(0.002)	0.019**<br>(0.007)		
Birth weight kg squared		-0.002*<br>(0.001)		
log(birth weight)			0.011*<br>(0.006)	
Birth weight above/below 2500 g.				0.009*<br>(0.004)
FE	Siblings	Siblings	Siblings	Siblings
Observations	147 888	147 888	147 888	147 888

Note: (Standard errors). Estimates and standard errors from OLS models for the association between minor traumatic brain injuries (mTBIs) and ever being convicted of a crime before age 20. Standard errors are clustered at the family level in all models. Control variables: sex and year of birth. Two-sided hypothesis tests, *** P < .001, **P < .01, *P < .05

© 2024 Blaabæk EH et al. *JAMA Pediatrics.*

eTable 3. Robustness to Birthweight Specifications OLS. Outcome: Ever Convicted of a Crime. Models With Twin Fixed Effects

	M1	M2	M3	M4
mTBI	-0.011<br>(0.012)	-0.011<br>(0.012)	-0.011<br>(0.012)	-0.010<br>(0.012)
Birth weight kg linear	0.013<br>(0.007)	0.058*<br>(0.027)		
Birth weight kg squared		-0.009<br>(0.005)		
log(birth weight)			0.041*<br>(0.017)	
Birth weight above/below 2500 g.				0.013<br>(0.007)
FE	Twins	Twins	Twins	Twins
Observations	11 688	11 688	11 668	11 688

Note: (Standard errors). Estimates and standard errors from OLS models for the association between minor traumatic brain injuries (mTBIs) and ever being convicted of a crime before age 20. Standard errors are clustered at the family level in all models. Control variables: sex and year of birth. Two-sided hypothesis tests, *** P < .001, **P < .01, *P < .05

eTable 4. Odds Ratios From logistic Regression Models for the Association between Minor Traumatic Brain Injury (mTBI; before age 10) and Ever Being Convicted of a Crime Before Age 20

	M1	M2	M3	M4	M5	M6
	Charged	Charged	Charged	Convicted	Convicted	Convicted
mTBI	1.285<br>[1.210 to 1.364]	0.969<br>[0.853 to 1.101]	0.939<br>[0.579 to 1.526]	1.271<br>[1.188 to 1.360]	0.947<br>[0.819 to 1.096]	0.856<br>[0.513 to 1.428]
Fixed effects	None	Siblings	Twins	None	Siblings	Twins
N	343 027	147 888	11 688	343 027	147 888	11 688

Note: Odds ratio point estimates and 95% confidence intervals for the association between minor traumatic brain injuries (mTBIs) and criminal charges and convictions. Standard errors are clustered at the family level in all models. Control variables used in all models: sex, year of birth.

© 2024 Blaabæk EH et al. *JAMA Pediatrics.*

eTable 5. Descriptive Statistics

	Total sample (N = 347 027)		Sibling sample (N = 147 888)		Twin sample (N = 11 688)	
Statistic	Mean	St. Dev.	Mean	St. Dev.	Mean	St. Dev.
Minor traumatic brain injury (mTBI)	0.04	0.19	0.04	0.19	0.04	0.20
Ever charged: all crime	0.06	0.24	0.06	0.24	0.05	0.22
Ever convicted: all crime	0.05	0.22	0.05	0.21	0.04	0.19
Ever charged: destructive crime	0.03	0.18	0.03	0.17	0.02	0.16
Ever convicted: destructive crime	0.02	0.15	0.02	0.14	0.02	0.13
Ever charged: violent crime	0.02	0.14	0.02	0.14	0.01	0.12
Ever convicted: violent crime	0.01	0.11	0.01	0.11	0.01	0.09
Number of crime charges	0.28	5.47	0.27	4.28	0.20	2.50
Number of crime convictions	0.08	0.45	0.08	0.45	0.06	0.41
Number of destructive crime charges	0.09	0.89	0.09	0.87	0.07	0.68
Number of destructive crime convictions	0.03	0.24	0.03	0.24	0.02	0.22
Number of violent crime charges	0.04	0.42	0.04	0.45	0.03	0.35
Number of violent crime convictions	0.02	0.16	0.02	0.16	0.01	0.13
Sex (ref = male)	0.49	0.50	0.49	0.50	0.49	0.50
Danish origin	0.95	0.22	0.94	0.23	0.97	0.18
Western origin	0.003	0.06	0.003	0.05	0.003	0.05
Non-Western origin	0.05	0.21	0.05	0.23	0.03	0.18
Mother: Less than high school	0.26	0.44	0.23	0.42	0.22	0.42
Mother: High school and vocational	0.51	0.50	0.52	0.50	0.52	0.50
Mother: College	0.23	0.42	0.25	0.43	0.26	0.44
Father: Less than high school	0.26	0.44	0.23	0.42	0.23	0.42
Father: High school and vocational	0.56	0.50	0.57	0.49	0.57	0.49
Father: College	0.18	0.39	0.20	0.40	0.19	0.40
Birth order: 1	0.49	0.50	0.38	0.49	0.28	0.45
Birth order: 2	0.37	0.48	0.46	0.50	0.45	0.50
Birth order: 3	0.12	0.32	0.12	0.32	0.20	0.40
Birth order: 4	0.02	0.15	0.03	0.16	0.05	0.22
Birth order: 5	0.01	0.09	0.01	0.10	0.02	0.12
Birth year: 1995	0.17	0.38	0.17	0.37	0.16	0.36
Birth year: 1996	0.17	0.37	0.16	0.37	0.17	0.38
Birth year: 1997	0.17	0.37	0.17	0.37	0.17	0.37
Birth year: 1998	0.16	0.37	0.17	0.38	0.17	0.37
Birth year: 1999	0.16	0.37	0.16	0.37	0.16	0.37
Birth year: 2000	0.17	0.37	0.17	0.38	0.17	0.38
Birth weight above 2500 g.	0.95	0.21	0.94	0.24	0.60	0.49

© 2024 Blaabæk EH et al. *JAMA Pediatrics.*

eTable 6. Association Between mTBI and the Number of ER Fracture/Sprain/Contusion Events

	M1<br>Total fracture events	M2<br>Total sprain events	M3<br>Total contusion events	M4<br>Total fracture events	M5<br>Total sprain events	M6<br>Total contusion events
mTBI	-0.007<br>(0.015)	0.009<br>(0.010)	0.040**<br>(0.013)	-0.069<br>(0.045)	-0.013<br>(0.029)	0.001<br>(0.040)
Fixed-effect	siblings	siblings	siblings	twins	twins	twins
observations	147 888	147 888	147 888	11 688	11 688	11 688

Note: Estimates from OLS fixed-effect models on the association between minor traumatic brain injuries (mTBIs) and the number of fracture/sprain/contusion events, wherehe injury involving mTBI is omitted from the count of events. Control variables: sex, birth year, mother's education, father's education, origin, birth order, birth weight. Standard errors are clustered at the family level in all models. Two-sided hypothesis tests, *** P < .001, **P < .01, *P < .05

© 2024 Blaabæk EH et al. *JAMA Pediatrics.*

eTable 7. OR Estimates From All logit Models on Ever Being Charged or Convicted of a Crime

	Outcome	Model	Fixed effect	Sample	Estimate	95% conf. interval
1	Charge - Crime	Ever - logit	No fixed effect	All	1.258	[1.185 to 1.336]
2	Charge - Crime	Ever - logit	No fixed effect	Males	1.277	[1.194 to 1.367]
3	Charge - Crime	Ever - logit	No fixed effect	Female	1.189	[1.046 to 1.351]
4	Charge - Crime	Ever - logit	Sibling fixed effect	All	0.968	[0.852 to 1.099]
5	Charge - Crime	Ever - logit	Sibling fixed effect	Males	1.077	[0.915 to 1.268]
6	Charge - Crime	Ever - logit	Sibling fixed effect	Female	0.846	[0.568 to 1.259]
7	Charge - Crime	Ever - logit	Twin fixed effect	All	0.940	[0.579 to 1.526]
8	Charge - Crime	Ever - logit	Twin fixed effect	Males	0.930	[0.547 to 1.584]
9	Conviction - Crime	Ever - logit	No fixed effect	All	1.241	[1.160 to 1.328]
10	Conviction - Crime	Ever - logit	No fixed effect	Males	1.250	[1.157 to 1.350]
11	Conviction - Crime	Ever - logit	No fixed effect	Female	1.205	[1.049 to 1.385]
12	Conviction - Crime	Ever - logit	Sibling fixed effect	All	0.945	[0.817 to 1.093]
13	Conviction - Crime	Ever - logit	Sibling fixed effect	Males	1.110	[0.915 to 1.347]
14	Conviction - Crime	Ever - logit	Sibling fixed effect	Female	0.900	[0.583 to 1.388]
15	Conviction - Crime	Ever - logit	Twin fixed effect	All	0.844	[0.505 to 1.411]
16	Conviction - Crime	Ever - logit	Twin fixed effect	Males	0.900	[0.526 to 1.542]

Note: Odds ratios and 95% confidence intervals for the association between mTBI and criminal charges and convictions from logistic-regression models (without fixed effects) and conditional logistic-regression models (with fixed effects) across sex at birth. Standard errors are clustered at the family level. Control variables include sex at birth, year of birth, birth weight, birth order, parent’s educational levels, parents’ origin.

© 2024 Blaabæk EH et al. *JAMA Pediatrics.*

eTable 8. IRR Estimates from Poisson Models on Crime Charges and Convictions

	Outcome	Model	Fixed effect	Sample	Estimate	95% conf. interval
1	Charge - Crime	Ever - Poisson	No fixed effect	All	1.239	[1.172 to 1.310]
2	Charge - Crime	Ever - Poisson	No fixed effect	Males	1.254	[1.178 to 1.334]
3	Charge - Crime	Ever - Poisson	No fixed effect	Female	1.184	[1.046 to 1.340]
4	Charge - Crime	Ever - Poisson	Sibling fixed effect	All	0.968	[0.854 to 1.098]
5	Charge - Crime	Ever - Poisson	Sibling fixed effect	Males	1.075	[0.916 to 1.262]
6	Charge - Crime	Ever - Poisson	Sibling fixed effect	Female	0.843	[0.567 to 1.254]
7	Charge - Crime	Ever - Poisson	Twin fixed effect	All	0.940	[0.578 to 1.528]
8	Charge - Crime	Ever - Poisson	Twin fixed effect	Males	0.930	[0.546 to 1.586]
9	Charge - Crime	Count - Poisson	No fixed effect	All	1.224	[1.024 to 1.464]
10	Charge - Crime	Count - Poisson	No fixed effect	Males	1.148	[0.990 to 1.331]
11	Charge - Crime	Count - Poisson	No fixed effect	Female	1.645	[0.849 to 3.187]
12	Charge - Crime	Count - Poisson	Sibling fixed effect	All	0.805	[0.627 to 1.035]
13	Charge - Crime	Count - Poisson	Sibling fixed effect	Males	0.937	[0.666 to 1.316]
14	Charge - Crime	Count - Poisson	Sibling fixed effect	Female	0.600	[0.328 to 1.098]
15	Charge - Crime	Count - Poisson	Twin fixed effect	All	0.479	[0.189 to 1.216]
16	Charge - Crime	Count - Poisson	Twin fixed effect	Males	0.335	[0.100 to 1.127]
17	Conviction - Crime	Ever - Poisson	No fixed effect	All	1.228	[1.152 to 1.309]
18	Conviction - Crime	Ever - Poisson	No fixed effect	Males	1.234	[1.148 to 1.327]
19	Conviction - Crime	Ever - Poisson	No fixed effect	Female	1.201	[1.049 to 1.376]
20	Conviction - Crime	Ever - Poisson	Sibling fixed effect	All	0.949	[0.822 to 1.096]
21	Conviction - Crime	Ever - Poisson	Sibling fixed effect	Males	1.114	[0.921 to 1.348]
22	Conviction - Crime	Ever - Poisson	Sibling fixed effect	Female	0.900	[0.583 to 1.390]
23	Conviction - Crime	Ever - Poisson	Twin fixed effect	All	0.844	[0.504 to 1.413]
24	Conviction - Crime	Ever - Poisson	Twin fixed effect	Males	0.900	[0.525 to 1.545]
25	Conviction - Crime	Count - Poisson	No fixed effect	All	1.282	[1.178 to 1.395]
26	Conviction - Crime	Count - Poisson	No fixed effect	Males	1.299	[1.178 to 1.432]
27	Conviction - Crime	Count - Poisson	No fixed effect	Female	1.201	[1.023 to 1.410]
28	Conviction - Crime	Count - Poisson	Sibling fixed effect	All	0.949	[0.794 to 1.135]
29	Conviction - Crime	Count - Poisson	Sibling fixed effect	Males	1.150	[0.893 to 1.482]
30	Conviction - Crime	Count - Poisson	Sibling fixed effect	Female	0.843	[0.503 to 1.412]
31	Conviction - Crime	Count - Poisson	Twin fixed effect	All	0.795	[0.453 to 1.394]
32	Conviction - Crime	Count - Poisson	Twin fixed effect	Males	0.616	[0.366 to 1.036]

Note: Incidence risk ratios and 95% confidence intervals for the association between mTBI and criminal charges and convictions (binary and count outcome) from Poisson models with and without fixed effects across sex at birth. Standard errors are clustered at the family level. Control variables include sex at birth, year of birth, birth weight, birth order, parent's educational levels, parents' origin.

© 2024 Blaabæk EH et al. *JAMA Pediatrics.*

eTable 9. Estimates From OLS Models on Crime Charges and Convictions

	Outcome	Model	Fixed effect	Sample	Estimate	95% conf. interval
1	Charge - Crime	Ever - OLS	No fixed effect	All	0.013	[0.009 to 0.017]
2	Charge - Crime	Ever - OLS	No fixed effect	Males	0.018	[0.013 to 0.024]
3	Charge - Crime	Ever - OLS	No fixed effect	Female	0.005	[0.001 to 0.009]
4	Charge - Crime	Ever - OLS	Sibling fixed effect	All	0.000	[-0.008 to 0.007]
5	Charge - Crime	Ever - OLS	Sibling fixed effect	Males	0.006	[-0.008 to 0.021]
6	Charge - Crime	Ever - OLS	Sibling fixed effect	Female	-0.004	[-0.016 to 0.007]
7	Charge - Crime	Ever - OLS	Twin fixed effect	All	-0.005	[-0.027 to 0.017]
8	Charge - Crime	Ever - OLS	Twin fixed effect	Males	-0.005	[-0.051 to 0.041]
9	Charge - Crime	Count - OLS	No fixed effect	All	0.002	[0.000 to 0.005]
10	Charge - Crime	Count - OLS	No fixed effect	Males	0.002	[0.000 to 0.005]
11	Charge - Crime	Count - OLS	No fixed effect	Female	0.002	[-0.002 to 0.006]
12	Charge - Crime	Count - OLS	Sibling fixed effect	All	-0.004	[-0.008 to 0.000]
13	Charge - Crime	Count - OLS	Sibling fixed effect	Males	-0.002	[-0.008 to 0.005]
14	Charge - Crime	Count - OLS	Sibling fixed effect	Female	-0.001	[-0.002 to 0.000]
15	Charge - Crime	Count - OLS	Twin fixed effect	All	-0.005	[-0.014 to 0.005]
16	Charge - Crime	Count - OLS	Twin fixed effect	Males	-0.017	[-0.044 to 0.011]
17	Conviction - Crime	Ever - OLS	No fixed effect	All	0.011	[0.007 to 0.015]
18	Conviction - Crime	Ever - OLS	No fixed effect	Males	0.015	[0.009 to 0.021]
19	Conviction - Crime	Ever - OLS	No fixed effect	Female	0.005	[0.001 to 0.009]
20	Conviction - Crime	Ever - OLS	Sibling fixed effect	All	-0.002	[-0.009 to 0.005]
21	Conviction - Crime	Ever - OLS	Sibling fixed effect	Males	0.008	[-0.006 to 0.022]
22	Conviction - Crime	Ever - OLS	Sibling fixed effect	Female	-0.003	[-0.015 to 0.009]
23	Conviction - Crime	Ever - OLS	Twin fixed effect	All	-0.009	[-0.030 to 0.012]
24	Conviction - Crime	Ever - OLS	Twin fixed effect	Males	-0.006	[-0.047 to 0.035]
25	Conviction - Crime	Count - OLS	No fixed effect	All	0.010	[0.006 to 0.014]
26	Conviction - Crime	Count - OLS	No fixed effect	Males	0.015	[0.009 to 0.022]
27	Conviction - Crime	Count - OLS	No fixed effect	Female	0.003	[0.000 to 0.007]
28	Conviction - Crime	Count - OLS	Sibling fixed effect	All	-0.003	[-0.011 to 0.005]
29	Conviction - Crime	Count - OLS	Sibling fixed effect	Males	0.009	[-0.007 to 0.025]
30	Conviction - Crime	Count - OLS	Sibling fixed effect	Female	-0.003	[-0.012 to 0.006]
31	Conviction - Crime	Count - OLS	Twin fixed effect	All	-0.007	[-0.028 to 0.014]
32	Conviction - Crime	Count - OLS	Twin fixed effect	Males	-0.024	[-0.058 to 0.010]

Note: Point estimates and 95% confidence intervals for the association between mTBI and criminal charges and convictions from OLS-regression models (with and without out fixed effects) across sex at birth. Standard errors are clustered at the family level. Control variables include sex at birth, year of birth, birth weight, birth order, parent's educational levels, parents' origin. All outcomes in OLS models are standardized to mean zero and standard deviation 1.

© 2024 Blaabæk EH et al. *JAMA Pediatrics.*

eTable 10. OR Estimates From logit Models on Ever Being Charged or Convicted of a Destructive Crime

	Outcome	Model	Fixed effect	Sample	Estimate	95% conf. interval
1	Charge - Destructive	Ever - logit	No fixed effect	All	1.303	[1.202 to 1.413]
2	Charge - Destructive	Ever - logit	No fixed effect	Males	1.291	[1.184 to 1.408]
3	Charge - Destructive	Ever - logit	No fixed effect	Female	1.380	[1.102 to 1.729]
4	Charge - Destructive	Ever - logit	Sibling fixed effect	All	0.943	[0.775 to 1.148]
5	Charge - Destructive	Ever - logit	Sibling fixed effect	Males	0.921	[0.736 to 1.152]
6	Charge - Destructive	Ever - logit	Sibling fixed effect	Female	0.599	[0.268 to 1.342]
7	Charge - Destructive	Ever - logit	Twin fixed effect	All	1.020	[0.482 to 2.160]
8	Charge - Destructive	Ever - logit	Twin fixed effect	Males	1.000	[0.453 to 2.206]
9	Conviction - Destructive	Ever - logit	No fixed effect	All	1.273	[1.152 to 1.407]
10	Conviction - Destructive	Ever - logit	No fixed effect	Males	1.263	[1.135 to 1.406]
11	Conviction - Destructive	Ever - logit	No fixed effect	Female	1.338	[1.010 to 1.774]
12	Conviction - Destructive	Ever - logit	Sibling fixed effect	All	1.057	[0.825 to 1.355]
13	Conviction - Destructive	Ever - logit	Sibling fixed effect	Males	1.182	[0.888 to 1.573]
14	Conviction - Destructive	Ever - logit	Sibling fixed effect	Female	0.695	[0.275 to 1.757]
15	Conviction - Destructive	Ever - logit	Twin fixed effect	All	1.069	[0.439 to 2.603]
16	Conviction - Destructive	Ever - logit	Twin fixed effect	Males	1.390	[0.545 to 3.547]

Note: Odds ratios and 95% confidence intervals for the association between mTBI and charges and convictions for destructive crime from logistic-regression models (without fixed effects) and conditional logistic-regression models (with fixed effects) across sex at birth. Standard errors are clustered at the family level. Control variables include sex at birth, year of birth, birth weight, birth order, parent's educational levels, parents' origin.

© 2024 Blaabæk EH et al. *JAMA Pediatrics.*

eTable 11. IRR Estimates From Poisson Models on Destructive Crime Charges and Convictions

	Outcome	Model	Fixed effect	Sample	Estimate	95% conf. interval
1	Charge - Destructive	Ever - Poisson	No fixed effect	All	1.287	[1.192 to 1.391]
2	Charge - Destructive	Ever - Poisson	No fixed effect	Males	1.274	[1.174 to 1.384]
3	Charge - Destructive	Ever - Poisson	No fixed effect	Female	1.377	[1.102 to 1.720]
4	Charge - Destructive	Ever - Poisson	Sibling fixed effect	All	0.949	[0.781 to 1.153]
5	Charge - Destructive	Ever - Poisson	Sibling fixed effect	Males	0.926	[0.742 to 1.155]
6	Charge - Destructive	Ever - Poisson	Sibling fixed effect	Female	0.600	[0.267 to 1.350]
7	Charge - Destructive	Ever - Poisson	Twin fixed effect	All	1.020	[0.48 0; 2.168]
8	Charge - Destructive	Ever - Poisson	Twin fixed effect	Males	1.000	[0.452 to 2.215]
9	Charge - Destructive	Count - Poisson	No fixed effect	All	1.305	[1.154 to 1.475]
10	Charge - Destructive	Count - Poisson	No fixed effect	Males	1.324	[1.162 to 1.509]
11	Charge - Destructive	Count - Poisson	No fixed effect	Female	1.101	[0.814 to 1.490]
12	Charge - Destructive	Count - Poisson	Sibling fixed effect	All	0.830	[0.619 to 1.112]
13	Charge - Destructive	Count - Poisson	Sibling fixed effect	Males	0.795	[0.549 to 1.153]
14	Charge - Destructive	Count - Poisson	Sibling fixed effect	Female	0.342	[0.114 to 1.028]
15	Charge - Destructive	Count - Poisson	Twin fixed effect	All	0.770	[0.354 to 1.674]
16	Charge - Destructive	Count - Poisson	Twin fixed effect	Males	0.699	[0.276 to 1.769]
17	Conviction - Destructive	Ever - Poisson	No fixed effect	All	1.264	[1.147 to 1.393]
18	Conviction - Destructive	Ever - Poisson	No fixed effect	Males	1.253	[1.130 to 1.390]
19	Conviction - Destructive	Ever - Poisson	No fixed effect	Female	1.336	[1.010 to 1.768]
20	Conviction - Destructive	Ever - Poisson	Sibling fixed effect	All	1.056	[0.825 to 1.351]
21	Conviction - Destructive	Ever - Poisson	Sibling fixed effect	Males	1.178	[0.887 to 1.564]
22	Conviction - Destructive	Ever - Poisson	Sibling fixed effect	Female	0.696	[0.273 to 1.771]
23	Conviction - Destructive	Ever - Poisson	Twin fixed effect	All	1.069	[0.436 to 2.619]
24	Conviction - Destructive	Ever - Poisson	Twin fixed effect	Males	1.390	[0.541 to 3.572]
25	Conviction - Destructive	Count - Poisson	No fixed effect	All	1.266	[1.128 to 1.422]
26	Conviction - Destructive	Count - Poisson	No fixed effect	Males	1.279	[1.129 to 1.448]
27	Conviction - Destructive	Count - Poisson	No fixed effect	Female	1.148	[0.863 to 1.528]
28	Conviction - Destructive	Count - Poisson	Sibling fixed effect	All	0.986	[0.759 to 1.280]
29	Conviction - Destructive	Count - Poisson	Sibling fixed effect	Males	1.095	[0.794 to 1.509]
30	Conviction - Destructive	Count - Poisson	Sibling fixed effect	Female	0.586	[0.213 to 1.612]
31	Conviction - Destructive	Count - Poisson	Twin fixed effect	All	0.688	[0.273 to 1.732]
32	Conviction - Destructive	Count - Poisson	Twin fixed effect	Males	0.760	[0.276 to 2.093]

Note: Incidence risk ratios and 95% confidence intervals for the association between mTBI and charges and convictions (binary and count outcome) for destructive crime from Poisson models with and without fixed effects across sex at birth. Standard errors are clustered at the family level. Control variables include sex at birth, year of birth, birth weight, birth order, parent’s educational levels, parents’ origin.

© 2024 Blaabæk EH et al. *JAMA Pediatrics.*

eTable 12. Estimates From OLS Models on Destructive Crime Charges and Convictions

	Outcome	Model	Fixed effect	Sample	Estimate	95% conf. interval
1	Charge - Destructive	Ever - OLS	No fixed effect	All	0.011	[0.007 to 0.015]
2	Charge - Destructive	Ever - OLS	No fixed effect	Males	0.016	[0.010 to 0.023]
3	Charge - Destructive	Ever - OLS	No fixed effect	Female	0.004	[0.001 to 0.007]
4	Charge - Destructive	Ever - OLS	Sibling fixed effect	All	-0.001	[-0.008 to 0.007]
5	Charge - Destructive	Ever - OLS	Sibling fixed effect	Males	-0.005	[-0.022 to 0.012]
6	Charge - Destructive	Ever - OLS	Sibling fixed effect	Female	-0.005	[-0.014 to 0.004]
7	Charge - Destructive	Ever - OLS	Twin fixed effect	All	-0.001	[-0.027 to 0.024]
8	Charge - Destructive	Ever - OLS	Twin fixed effect	Males	0	[-0.053 to 0.053]
9	Charge - Destructive	Count - OLS	No fixed effect	All	0.006	[0.003 to 0.010]
10	Charge - Destructive	Count - OLS	No fixed effect	Males	0.011	[0.005 to 0.016]
11	Charge - Destructive	Count - OLS	No fixed effect	Female	0	[-0.001 to 0.002]
12	Charge - Destructive	Count - OLS	Sibling fixed effect	All	-0.006	[-0.014 to 0.002]
13	Charge - Destructive	Count - OLS	Sibling fixed effect	Males	-0.011	[-0.028 to 0.006]
14	Charge - Destructive	Count - OLS	Sibling fixed effect	Female	-0.002	[-0.005 to 0.001]
15	Charge - Destructive	Count - OLS	Twin fixed effect	All	-0.002	[-0.022 to 0.018]
16	Charge - Destructive	Count - OLS	Twin fixed effect	Males	-0.012	[-0.049 to 0.025]
17	Conviction - Destructive	Ever - OLS	No fixed effect	All	0.009	[0.005 to 0.012]
18	Conviction - Destructive	Ever - OLS	No fixed effect	Males	0.012	[0.006 to 0.019]
19	Conviction - Destructive	Ever - OLS	No fixed effect	Female	0.003	[0.000 to 0.006]
20	Conviction - Destructive	Ever - OLS	Sibling fixed effect	All	0.001	[-0.007 to 0.009]
21	Conviction - Destructive	Ever - OLS	Sibling fixed effect	Males	0.010	[-0.008 to 0.027]
22	Conviction - Destructive	Ever - OLS	Sibling fixed effect	Female	-0.002	[-0.012 to 0.007]
23	Conviction - Destructive	Ever - OLS	Twin fixed effect	All	0.002	[-0.023 to 0.027]
24	Conviction - Destructive	Ever - OLS	Twin fixed effect	Males	0.019	[-0.034 to 0.072]
25	Conviction - Destructive	Count - OLS	No fixed effect	All	0.007	[0.003 to 0.011]
26	Conviction - Destructive	Count - OLS	No fixed effect	Males	0.012	[0.005 to 0.018]
27	Conviction - Destructive	Count - OLS	No fixed effect	Female	0.001	[-0.001 to 0.003]
28	Conviction - Destructive	Count - OLS	Sibling fixed effect	All	-0.003	[-0.011 to 0.005]
29	Conviction - Destructive	Count - OLS	Sibling fixed effect	Males	0.004	[-0.013 to 0.022]
30	Conviction - Destructive	Count - OLS	Sibling fixed effect	Female	-0.002	[-0.009 to 0.004]
31	Conviction - Destructive	Count - OLS	Twin fixed effect	All	-0.011	[-0.034 to 0.013]
32	Conviction - Destructive	Count - OLS	Twin fixed effect	Males	-0.011	[-0.068 to 0.045]

Note: Point estimates and 95% confidence intervals for the association between mTBI and charges and convictions for destructive crime from OLS-regression models (with and without out fixed effects) across sex at birth. Standard errors are clustered at the family level. Control variables include sex at birth, year of birth, birth weight, birth order, parent's educational levels, parents' origin. All outcomes in OLS models are standardized to mean 0 and standard deviation 1.

© 2024 Blaabæk EH et al. *JAMA Pediatrics.*

eTable 13. OR Estimates From logit Models on Ever Being Charged or Convicted of a Violent Crime

	Outcome	Model	Fixed effect	Sample	Estimate	95% conf. interval
1	Charge - Violent	Ever - logit	No fixed effect	All	1.383	[1.245 to 1.535]
2	Charge - Violent	Ever - logit	No fixed effect	Males	1.375	[1.227 to 1.539]
3	Charge - Violent	Ever - logit	No fixed effect	Female	1.422	[1.083 to 1.866]
4	Charge - Violent	Ever - logit	Sibling fixed effect	All	0.928	[0.727 to 1.185]
5	Charge - Violent	Ever - logit	Sibling fixed effect	Males	0.940	[0.708 to 1.248]
6	Charge - Violent	Ever - logit	Sibling fixed effect	Female	0.666	[0.203 to 2.185]
7	Charge - Violent	Ever - logit	Twin fixed effect	All	1.996	[0.720 to 5.532]
8	Charge - Violent	Ever - logit	Twin fixed effect	Males	1.392	[0.549 to 3.529]
9	Conviction - Violent	Ever - logit	No fixed effect	All	1.350	[1.186 to 1.536]
10	Conviction - Violent	Ever - logit	No fixed effect	Males	1.365	[1.188 to 1.570]
11	Conviction - Violent	Ever - logit	No fixed effect	Female	1.252	[0.882 to 1.776]
12	Conviction - Violent	Ever - logit	Sibling fixed effect	All	1.105	[0.808 to 1.510]
13	Conviction - Violent	Ever - logit	Sibling fixed effect	Males	1.118	[0.770 to 1.625]
14	Conviction - Violent	Ever - logit	Sibling fixed effect	Female	0.649	[0.165 to 2.554]
15	Conviction - Violent	Ever - logit	Twin fixed effect	All	2.142	[0.604 to 7.598]
16	Conviction - Violent	Ever - logit	Twin fixed effect	Males	1.646	[0.473 to 5.728]

Note: Odds ratios and 95% confidence intervals for the association between mTBI and charges and convictions for violent crime from logistic-regression models (without fixed effects) and conditional logistic-regression models (with fixed effects) across sex at birth. Standard errors are clustered at the family level. Control variables include sex at birth, year of birth, birth weight, birth order, parent’s educational levels, parents’ origin.

© 2024 Blaabæk EH et al. *JAMA Pediatrics.*

eTable 14. IRR Estimates from Poisson Models on Violent Crime Charges or Convictions

	Outcome	Model	Fixed effect	Sample	Estimate	95% conf. interval
1	Charge - Violent	Ever - Poisson	No fixed effect	All	1.368	[1.237 to 1.514]
2	Charge - Violent	Ever - Poisson	No fixed effect	Males	1.358	[1.218 to 1.515]
3	Charge - Violent	Ever - Poisson	No fixed effect	Female	1.419	[1.083 to 1.858]
4	Charge - Violent	Ever - Poisson	Sibling fixed effect	All	0.936	[0.734 to 1.194]
5	Charge - Violent	Ever - Poisson	Sibling fixed effect	Males	0.952	[0.718 to 1.261]
6	Charge - Violent	Ever - Poisson	Sibling fixed effect	Female	0.664	[0.200 to 2.205]
7	Charge - Violent	Ever - Poisson	Twin fixed effect	All	1.996	[0.714 to 5.580]
8	Charge - Violent	Ever - Poisson	Twin fixed effect	Males	1.392	[0.544 to 3.560]
9	Charge - Violent	Count - Poisson	No fixed effect	All	1.285	[1.112 to 1.484]
10	Charge - Violent	Count - Poisson	No fixed effect	Males	1.308	[1.118 to 1.530]
11	Charge - Violent	Count - Poisson	No fixed effect	Female	1.092	[0.798 to 1.494]
12	Charge - Violent	Count - Poisson	Sibling fixed effect	All	0.717	[0.504 to 1.019]
13	Charge - Violent	Count - Poisson	Sibling fixed effect	Males	0.690	[0.436 to 1.091]
14	Charge - Violent	Count - Poisson	Sibling fixed effect	Female	0.364	[0.074 to 1.797]
15	Charge - Violent	Count - Poisson	Twin fixed effect	All	0.964	[0.376 to 2.471]
16	Charge - Violent	Count - Poisson	Twin fixed effect	Males	0.674	[0.275 to 1.652]
17	Conviction - Violent	Ever - Poisson	No fixed effect	All	1.342	[1.182 to 1.523]
18	Conviction - Violent	Ever - Poisson	No fixed effect	Males	1.356	[1.183 to 1.554]
19	Conviction - Violent	Ever - Poisson	No fixed effect	Female	1.250	[0.883 to 1.771]
20	Conviction - Violent	Ever - Poisson	Sibling fixed effect	All	1.108	[0.811 to 1.513]
21	Conviction - Violent	Ever - Poisson	Sibling fixed effect	Males	1.125	[0.776 to 1.630]
22	Conviction - Violent	Ever - Poisson	Sibling fixed effect	Female	0.650	[0.162 to 2.604]
23	Conviction - Violent	Ever - Poisson	Twin fixed effect	All	2.142	[0.595 to 7.721]
24	Conviction - Violent	Ever - Poisson	Twin fixed effect	Males	1.646	[0.464 to 5.835]
25	Conviction - Violent	Count - Poisson	No fixed effect	All	1.278	[1.109 to 1.472]
26	Conviction - Violent	Count - Poisson	No fixed effect	Males	1.304	[1.119 to 1.520]
27	Conviction - Violent	Count - Poisson	No fixed effect	Female	1.095	[0.769 to 1.561]
28	Conviction - Violent	Count - Poisson	Sibling fixed effect	All	1.026	[0.749 to 1.406]
29	Conviction - Violent	Count - Poisson	Sibling fixed effect	Males	1.057	[0.716 to 1.560]
30	Conviction - Violent	Count - Poisson	Sibling fixed effect	Female	0.612	[0.145 to 2.579]
31	Conviction - Violent	Count - Poisson	Twin fixed effect	All	1.244	[0.348 to 4.441]
32	Conviction - Violent	Count - Poisson	Twin fixed effect	Males	0.978	[0.273 to 3.502]

Note: Incidence risk ratios and 95% confidence intervals for the association between mTBI and charges and convictions (binary and count outcome) for violent crime from Poisson models with and without fixed effects across sex at birth. Standard errors are clustered at the family level. Control variables include sex at birth, year of birth, birth weight, birth order, parent's educational levels, parents' origin.

© 2024 Blaabæk EH et al. *JAMA Pediatrics.*

eTable 15. Estimates From OLS Models on Violent Crime Convictions

	Outcome	Model	FE	Sample	Estimate	95% conf. interval
1	Charge - Violent	Ever - OLS	No fixed effect	All	0.011	[0.007 to 0.015]
2	Charge - Violent	Ever - OLS	No fixed effect	Males	0.015	[0.009 to 0.022]
3	Charge - Violent	Ever - OLS	No fixed effect	Female	0.004	[0.000 to 0.007]
4	Charge - Violent	Ever - OLS	Sibling fixed effect	All	-0.002	[-0.010 to 0.006]
5	Charge - Violent	Ever - OLS	Sibling fixed effect	Males	-0.002	[-0.020 to 0.017]
6	Charge - Violent	Ever - OLS	Sibling fixed effect	Female	-0.003	[-0.012 to 0.006]
7	Charge - Violent	Ever - OLS	Twin fixed effect	All	0.015	[-0.010 to 0.040]
8	Charge - Violent	Ever - OLS	Twin fixed effect	Males	0.020	[-0.036 to 0.077]
9	Charge - Violent	Count - OLS	No fixed effect	All	0.006	[0.002 to 0.009]
10	Charge - Violent	Count - OLS	No fixed effect	Males	0.009	[0.003 to 0.015]
11	Charge - Violent	Count - OLS	No fixed effect	Female	0.000	[-0.001 to 0.002]
12	Charge - Violent	Count - OLS	Sibling fixed effect	All	-0.008	[-0.017 to 0.001]
13	Charge - Violent	Count - OLS	Sibling fixed effect	Males	-0.014	[-0.035 to 0.006]
14	Charge - Violent	Count - OLS	Sibling fixed effect	Female	-0.003	[-0.009 to 0.003]
15	Charge - Violent	Count - OLS	Twin fixed effect	All	0.000	[-0.022 to 0.022]
16	Charge - Violent	Count - OLS	Twin fixed effect	Males	-0.013	[-0.048 to 0.022]
17	Conviction - Violent	Ever - OLS	No fixed effect	All	0.008	[0.004 to 0.012]
18	Conviction - Violent	Ever - OLS	No fixed effect	Males	0.012	[0.006 to 0.019]
19	Conviction - Violent	Ever - OLS	No fixed effect	Female	0.002	[-0.001 to 0.005]
20	Conviction - Violent	Ever - OLS	Sibling fixed effect	All	0.002	[-0.006 to 0.010]
21	Conviction - Violent	Ever - OLS	Sibling fixed effect	Males	0.008	[-0.011 to 0.026]
22	Conviction - Violent	Ever - OLS	Sibling fixed effect	Female	-0.001	[-0.011 to 0.008]
23	Conviction - Violent	Ever - OLS	Twin fixed effect	All	0.015	[-0.010 to 0.040]
24	Conviction - Violent	Ever - OLS	Twin fixed effect	Males	0.025	[-0.035 to 0.085]
25	Conviction - Violent	Count - OLS	No fixed effect	All	0.006	[0.002 to 0.010]
26	Conviction - Violent	Count - OLS	No fixed effect	Males	0.010	[0.003 to 0.016]
27	Conviction - Violent	Count - OLS	No fixed effect	Female	0.001	[-0.002 to 0.003]
28	Conviction - Violent	Count - OLS	Sibling fixed effect	All	-0.002	[-0.011 to 0.006]
29	Conviction - Violent	Count - OLS	Sibling fixed effect	Males	0.003	[-0.014 to 0.021]
30	Conviction - Violent	Count - OLS	Sibling fixed effect	Female	-0.001	[-0.009 to 0.007]
31	Conviction - Violent	Count - OLS	Twin fixed effect	All	-0.001	[-0.027 to 0.025]
32	Conviction - Violent	Count - OLS	Twin fixed effect	Males	0.000	[-0.054 to 0.054]

Note: Point estimates and 95% confidence intervals for the association between mTBI and charges and convictions for destructive crime from OLS-regression models (with and without out fixed effects) across sex at birth. Standard errors are clustered at the family level. Control variables include sex at birth, year of birth, birth weight, birth order, parent's educational levels, parents' origin. All outcomes in OLS models are standardized to mean 0 and standard deviation 1.

© 2024 Blaabæk EH et al. *JAMA Pediatrics.*

eFigure 1. mTBI Rates by Age and Sex.

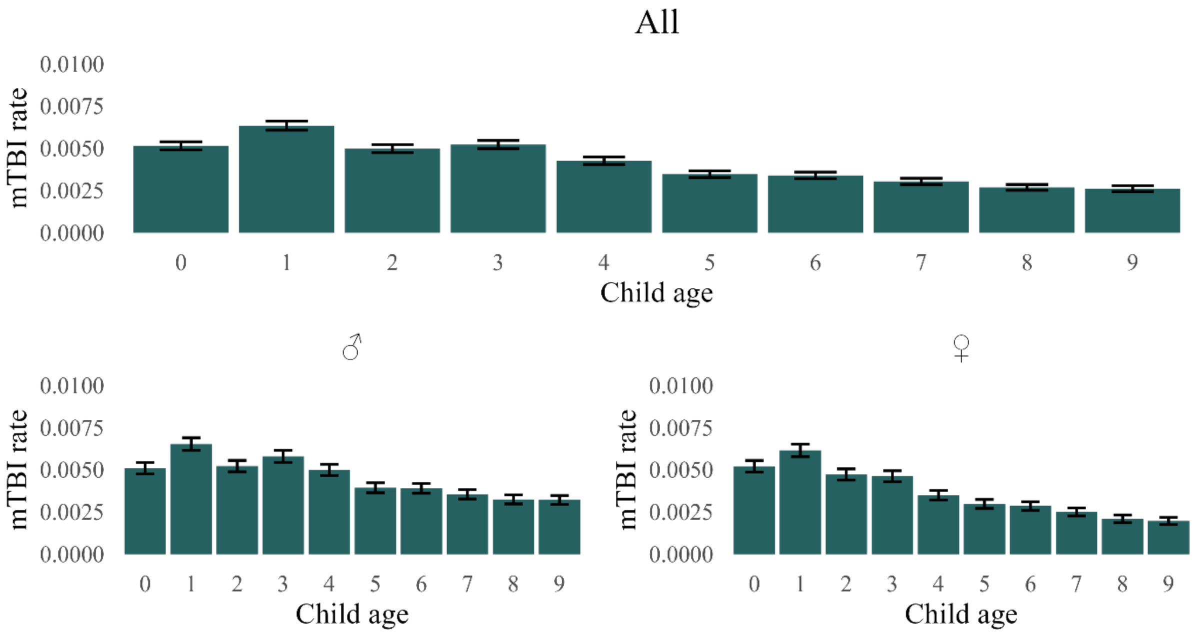

Note: Error bars represent 95% confidence intervals

© 2024 Blaabæk EH et al. *JAMA Pediatrics.*

eFigure 2. Estimated Associations (OLS Models on Binary Outcome) between mTBI (Before Age 10) and Ever Being Charged or Convicted of Criminal Offenses (Age 15 to 20) by Crime Type and Sex Across Preregistered Models

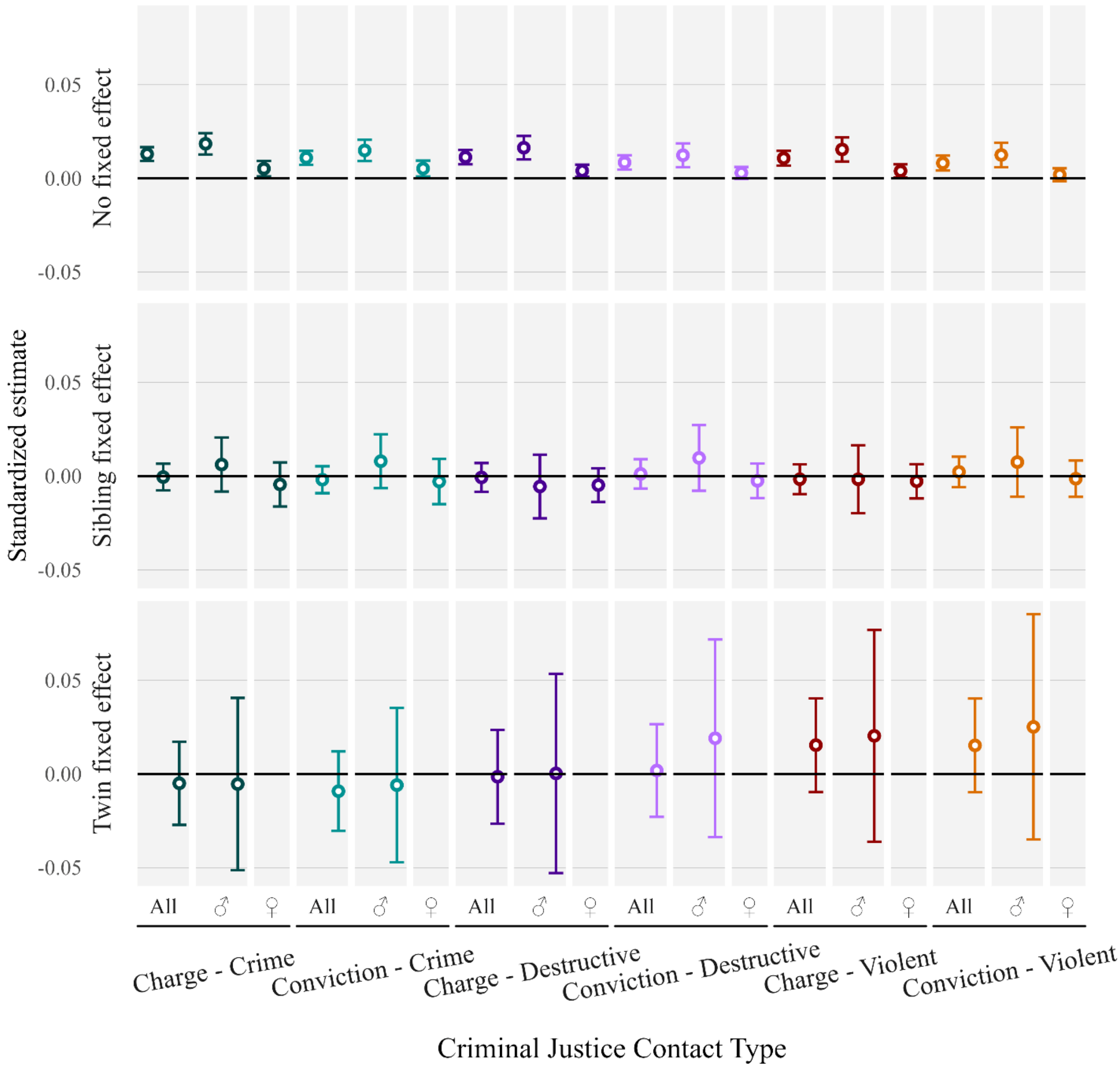

Note: OLS point estimates and 95% confidence intervals for the association between mTBI and criminal charges and convictions across three model specifications and nested samples. Results for female in twin fixed-effects models are masked due to insufficient cell size (confidentiality constraint imposed by Statistics Denmark). Confidence intervals without top bar exceed y-axis range.Outcomes are standardized to mean 0 and standard variation 1.

© 2024 Blaabæk EH et al. *JAMA Pediatrics.*

eFigure 3. Estimated Associations (OLS Models) between mTBI (Before Age 10) and Number of Criminal Charges and Convictions (Age 15 to 20) by Crime Type and Sex Across Preregistered Models

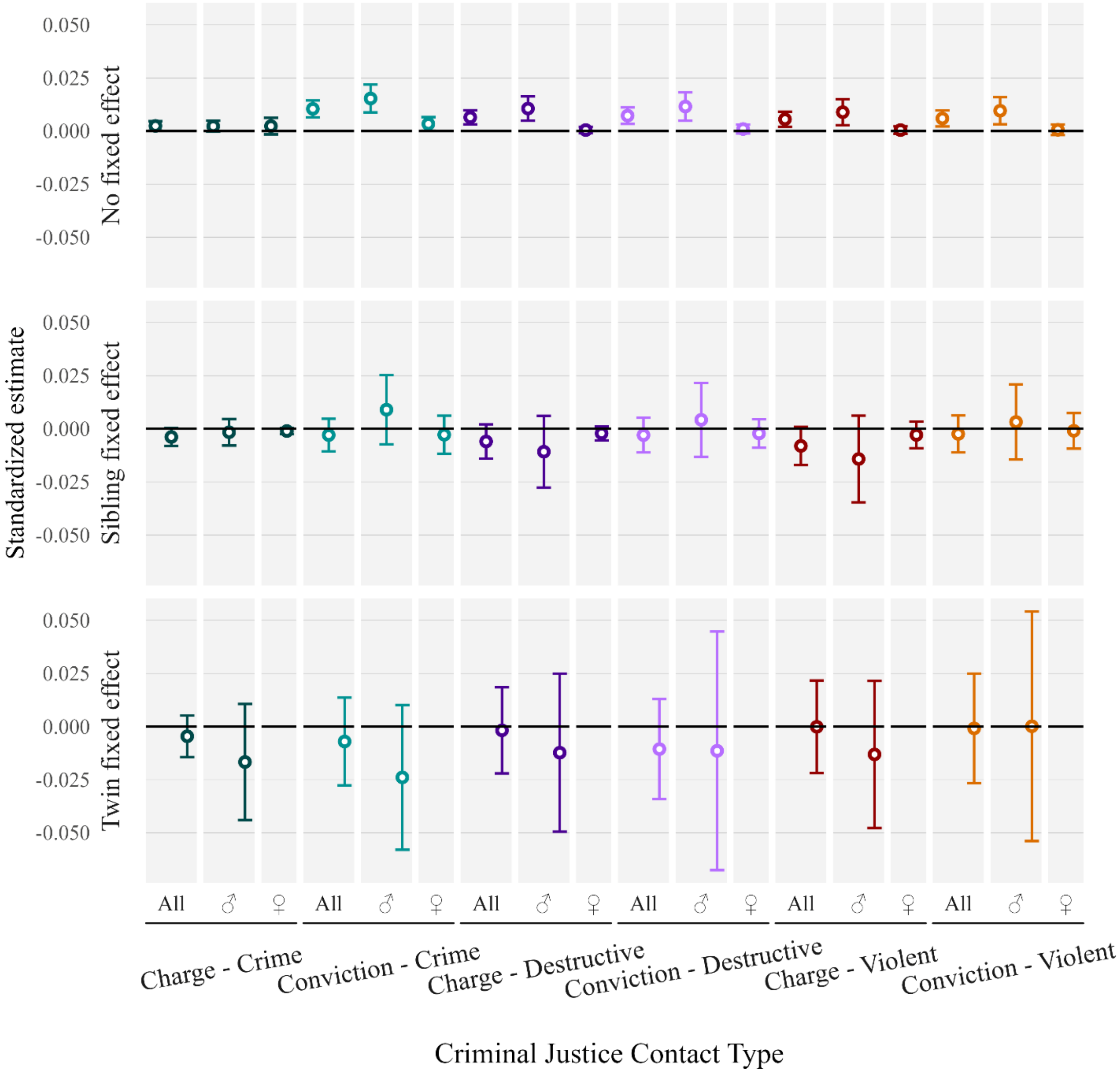

Note: OLS point estimates and 95% confidence intervals for the association between mTBI and criminal charges and convictions across three model specifications and nested samples. Results for female in twin fixed-effects models are masked due to insufficient cell size (confidentiality constraint imposed by Statistics Denmark). Confidence intervals without top bar exceed y-axis range. Outcomes are standardized to mean 0 and standard variation 1.

© 2024 Blaabæk EH et al. *JAMA Pediatrics.*

eFigure 4. Estimated Associations (Incidence Rate Ratios From Poisson Models) between mTBI (Before Age 10) and Ever Being Charged or Convicted of Criminal Offenses (Age 15 to 20) by Crime Type and Sex Across Preregistered Models

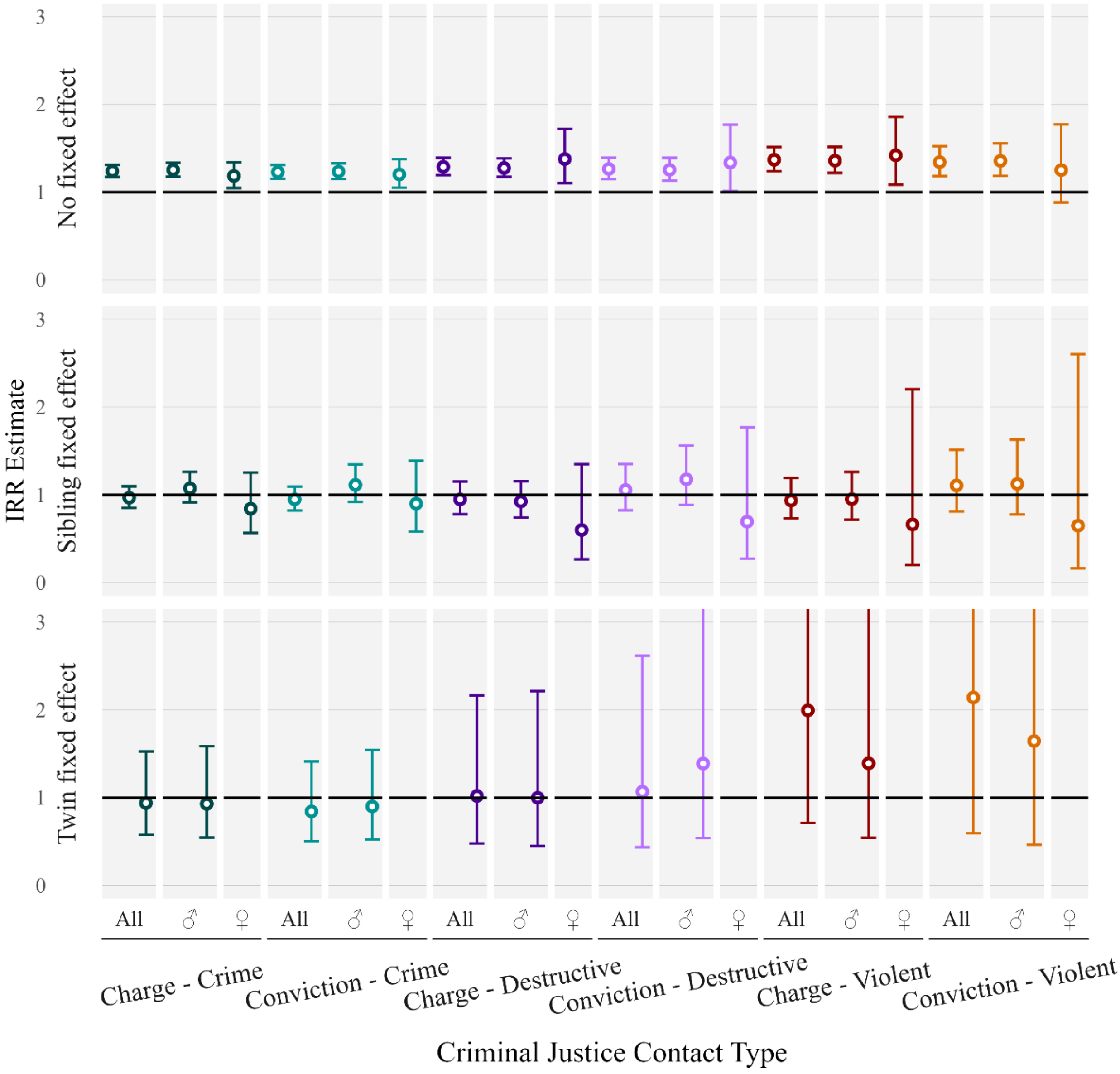

Note: IRR ratio point estimates and 95% confidence intervals for the association between mTBI and criminal charges and convictions across three model specifications and nested samples. Results for female in twin fixed-effects models are masked due to insufficient cell size (confidentiality constraint imposed by Statistics Denmark). Confidence intervals without top bar exceed y-axis range.

© 2024 Blaabæk EH et al. *JAMA Pediatrics.*

eFigure 5. Estimated Associations (Incidence Rate Ratios From Poisson Models) between mTBI (Before Age 10) and Number of Criminal Charges and Convictions (Age 15 to 20) by Crime Type and Sex Across Preregistered Models

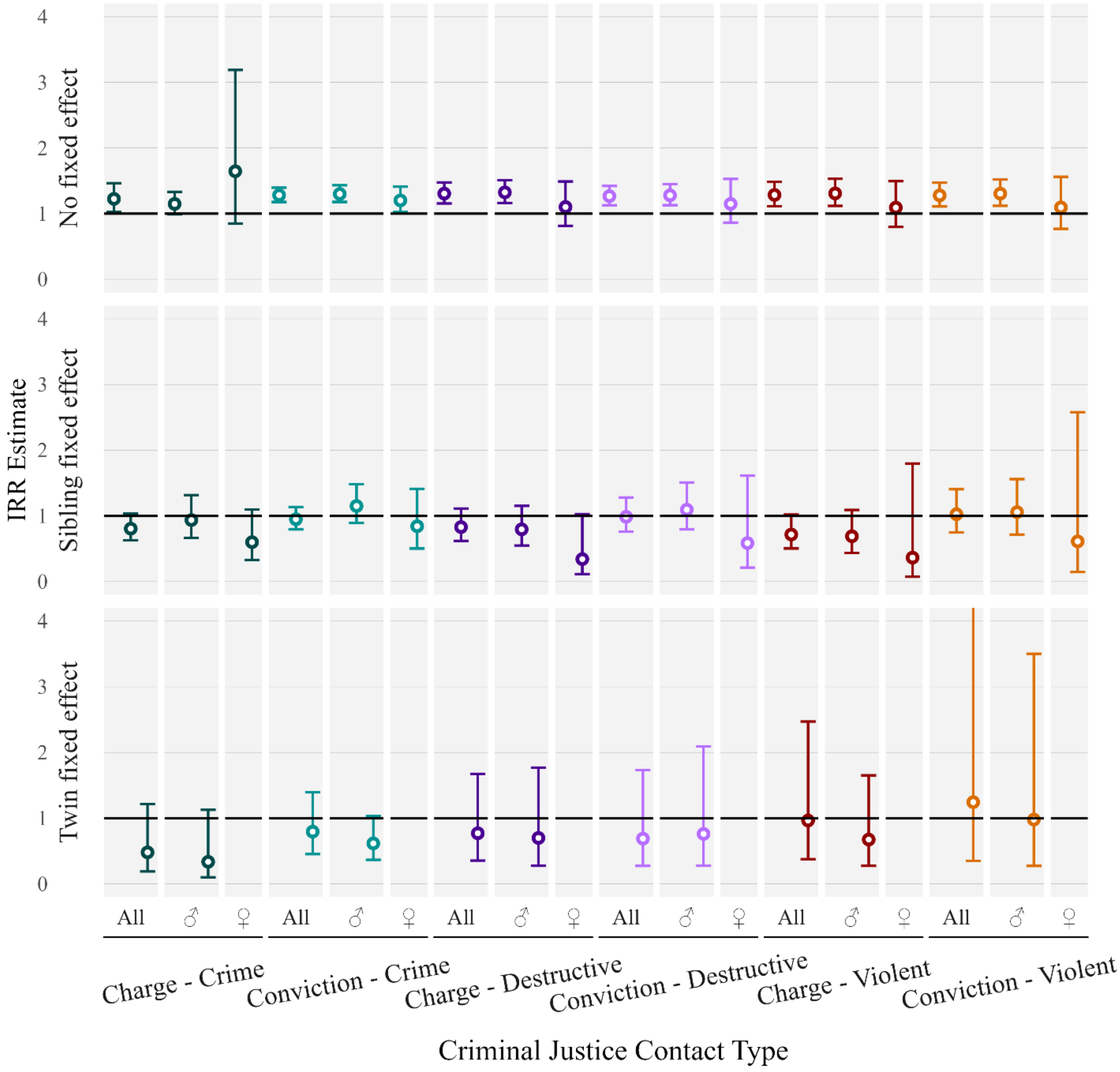

Note: IRR point estimates and 95% confidence intervals for the association between mTBI and criminal charges and convictions across three model specifications and nested samples. Results for female in twin fixed-effects models are masked due to insufficient cell size (confidentiality constraint imposed by Statistics Denmark). Confidence intervals without top bar exceed y-axis range.

© 2024 Blaabæk EH et al. *JAMA Pediatrics.*
