# Supplementary material for: Mild Traumatic Brain Injury and Criminal Charges and Convictions in Mid and Late Adolescence
Source: JAMA Pediatr. 2024 Sep 30;178(11):1164–71. doi: 10.1001/jamapediatrics.2024.3452 (PMC11443431; doi:10.1001/jamapediatrics.2024.3452)
Supplement: Supplement 2. — Data sharing statement [file jamapediatr-e243452-s002.pdf]

Data Sharing Statement

Blaabæk. Mild Traumatic Brain Injury and Criminal Charges and Convictions in Mid and Late Adolescence. *JAMA Pediatr*. Published September 30, 2024.
doi:10.1001/jamapediatrics.2024.3452

Data
Data available: No

Additional Information
Explanation for why data not available: The information used in the analysis combines several Danish administrative registers (as described in the paper). The data use is subject to the European Union's General Data Protection Regulation (GDPR) per Danish regulations from May 2018. The data are physically stored on computers at Statistics Denmark and, due to security considerations, may not be transferred to computers outside Statistics Denmark. Researchers interested in obtaining access to the register data employed in this paper are required to submit a written application to gain approval from Statistics Denmark. The application must include a detailed description of the proposed project, its purpose, and its social contribution as well as a description of the required datasets, variables, and analysis population. Applications can be submitted by researchers who are affiliated with Danish institutions accepted by Statistics Denmark or by researchers outside of Denmark who collaborate with researchers affiliated with these institutions. All coding files are available at https://osf.io/x27k3/?view_only=c5c684e0aaca4b8892e06e432453d01b
